# Parent–Infant Skin-to-Skin Contact and Stress Regulation: A Systematic Review of the Literature

**DOI:** 10.3390/ijerph18094695

**Published:** 2021-04-28

**Authors:** Chiara Ionio, Giulia Ciuffo, Marta Landoni

**Affiliations:** CRIdee, Psychology Department, Università Cattolica, del Sacro Cuore, 20123 Milan, Italy; giulia.ciuffo01@icatt.it (G.C.); marta.landoni@unicatt.it (M.L.)

**Keywords:** skin-to-skin contact, kangaroo care, stress, sympathetic nervous system, cortisol, newborn, systematic review

## Abstract

Several studies have focused on neonatal maternal separation (MS) to investigate behavioural and neuroendocrine reactions to lack of contact, but only a few have focused on early separation in the first days or weeks after birth. This literature review investigates the vital importance of contact and touch by exploring how skin-to-skin contact (SSC) regulates stress in the mother–infant relationship. Various databases such as PubMed, Scopus, and ScienceDirect were searched for literature published between 2015 and 2020. From 1141 articles, 22 were declared eligible. The reviewed articles showed how SSC regulates child stress by biological indicators such as the autonomic nervous system (ANS), heart rate variability (HRV), cortisol, and oxytocin. This research concludes the importance of SSC for stress regulation, especially during the COVID-19 pandemic. With no research to date indicating a possible risk of neonatal COVID-19 transmission following SSC, SSC should continue to be practiced for all women, as recommended by the WHO.

## 1. Introduction

In recent years, new knowledge in developmental psychology and infant mental health has confirmed the essential regulatory function of maternal presence [1]. In neuroscience, advanced imaging and validated autonomic nervous system (ANS) measures have provided an enhanced understanding of the interrelationship between the mother and the child’s ANS [2,3].

Porges and Furman [4] were the first authors to describe the evolutionary origin of the ANS. Indeed, the earliest version of the ANS was a visceral system, the “reptilian” brain composed of a parasympathetic nervous system (PSNS). The mammalian brain’s advance was the sympathetic nervous system (SNS), characterized by quick freezing, fighting, or flying [4]. However, studies have indicated how the SNS is also involved in homeostasis, even in favourable circumstances, and how it works very closely with the PSNS [4].

Furthermore, humans developed a new myelinated vagal system for prompt and precise control, connected to the cardiovascular and respiratory systems [5]. This is of particular importance for newborns. Studies have demonstrated how the old unmyelinated vagus could be mature and operative at 28 weeks. Babies born before that time could have severely impaired autonomic function. On the other hand, for babies born after 28 weeks, the PSNS could be considered relatively robust but immature until 46 to 48 weeks [6]. In particular, at 28 weeks, the response to stress for preterm babies is essentially a “reptilian” dissociation behaviour, stress-determined response [6]. These insights clearly explained how the mother’s constant and uninterrupted physical presence is necessary throughout early childhood and beyond [7].

Several studies have focused on neonatal maternal separation (MS) to explore the behavioural and neuroendocrine reaction to lack of contact [8], but only a few of them concentrated on early separation in the first days or weeks after birth [9].

Harlow performed the first significant experiments on maternal deprivation in monkeys in the 1950s and 1970s. Afterwards, Suomi conducted similar maternal deprivation experiments on newborn monkeys [10,11,12,13,14], which showed how the separation of primate infants from their mothers could lead to disastrous effects on their following development and behaviour. 

Considering these notions, the critical role of contact in early life days is evident.

In hospitals across the world, the practice of skin-to-skin contact (SSC) has recently started to gain more attention and importance. Early mother–infant SSC (also known as kangaroo care [KC]) is a procedure characterized by the contact of the naked neonate prone on the mother’s bare chest immediately after birth [15]. In the 1970s, in Bogota, the advantages of early mother–infant contact were recognized when only a few incubators were available to care for low-birthweight infants. In 2003, the World Health Organization (WHO) designated KC as the most effective method to maintain body temperature, stimulate senses, and provide maternal love [16].

Several studies have demonstrated the beneficial effects of SSC practice on preterm and full-term infants (e.g., enhancing the physiological, emotional, and cognitive regulatory processes) [17]. A meta-analysis by Moore et al. [17] highlighted the beneficial outcomes of SSC of breastfeeding for one to four months after birth on blood glucose, infant crying, and infant temperature control. SSC was also connected with lower salivary cortisol, lower heart rate, better sleep–wake cycles, and enhanced mood [17]. However, to date, the underlying neurobiological mechanisms are still not understood. 

One explanation could be that intimate interaction and contact between mother and child addressed the infant’s essential biological needs (such as warmth, touch, and smell) and hence regulated infant physiology, including the hypothalamic–pituitary–adrenal (HPA) axis [18]. 

Indeed, several studies have demonstrated how contact and touch are associated with the activity of the HPA axis, particularly during critical periods of development [19,20]. This effect is especially pronounced in the bond between the mother and the child; maternal contact has been found to reduce the infant’s physiological stress [21].

The HPA axis is stimulated when threatened with stressors, and cortisol is released [22]. Although this rise helps to cope with a stressor, reactions to frequent and chronic stress can affect the body and are linked to physical and mental health issues [23,24]. SSC can enable the infant to regulate the physiology of cortisol. Studies have demonstrated how a 20-min period of mother–infant SSC was combined with a substantial reduction in preterm cortisol concentrations [25]. These results suggest that SSC might be a successful intervention to reduce infant stress [26].

Nowadays, contact is even more significant and a priority worldwide due to the COVID-19 pandemic. The novel coronavirus, COVID-19, was first reported in Wuhan, China, and was declared a pandemic by the WHO between 2019 and 2020 [27]. Indeed, it quickly became evident that COVID-19 spread rapidly between people in close contact [28]. In attempts to stem the pandemic, public health campaigns about the importance of adopting physical distance were consistent around the world.

Furthermore, the COVID-19-related restrictions on hospital procedures caused additional distress for pregnant women; in many countries, women were requested to attend all prenatal appointments alone, and in some countries, women were even asked to be alone during birth. In sum, this lack of contact could elevate the level of prenatal psychological distress for both women and their babies.

Indeed, as previously seen, touch and contact are fundamental parts of the human experience. Both are related to socio-emotional, physical, cognitive, and neurological development in childhood and necessary forms of non-biological communication throughout life [29]. 

When touch is limited or eliminated, it could lead to developing what is known as touch starvation [30] or touch hunger [31] connected to adverse physical and psychological outcomes.

The current review aims to investigate and remind people of the vital importance of contact and touch. 

In particular, we aim to answer the following question: “How does SSC regulate stress in an infant–parent relationship?”

In the literature, there is evidence that stimulation of specific receptors in the skin and mother–child contact increase infant vagal activity [32]. Thus, through decreased vagal tone, which dysregulates the HPA axis, a lack of caregiver touch could lead to adverse mental and physical results [33,34]. 

## 2. Materials and Methods

To answer the research question, we followed the Preferred Reporting Items for Systematic Review and Meta-Analysis (PRISMA) guidelines in the current review [35].

PubMed, Scopus, and ScienceDirect were used as databases to identify articles using the following research string: “skin to skin contact OR kangaroo care OR infant touch AND stress AND sympathetic nervous system OR autonomic nervous OR cortisol AND newborn OR infant AND co-regulation in the title/abstract”. Records were considered eligible if articles were empirical research and peer reviewed, published in indexed scientific journals or pre-printed, written in English, published between 2015 and 2020, and answered the research question.

Two authors independently completed an inclusion/exclusion checklist while screening the titles, keywords, and abstracts of the primary search results to ensure reliability. 

The search yielded 1141 articles, of which 13 were removed as duplicates. Eleven articles were added manually. After screening for abstracts and titles, 22 articles were declared eligible. 

**Figure 1:**
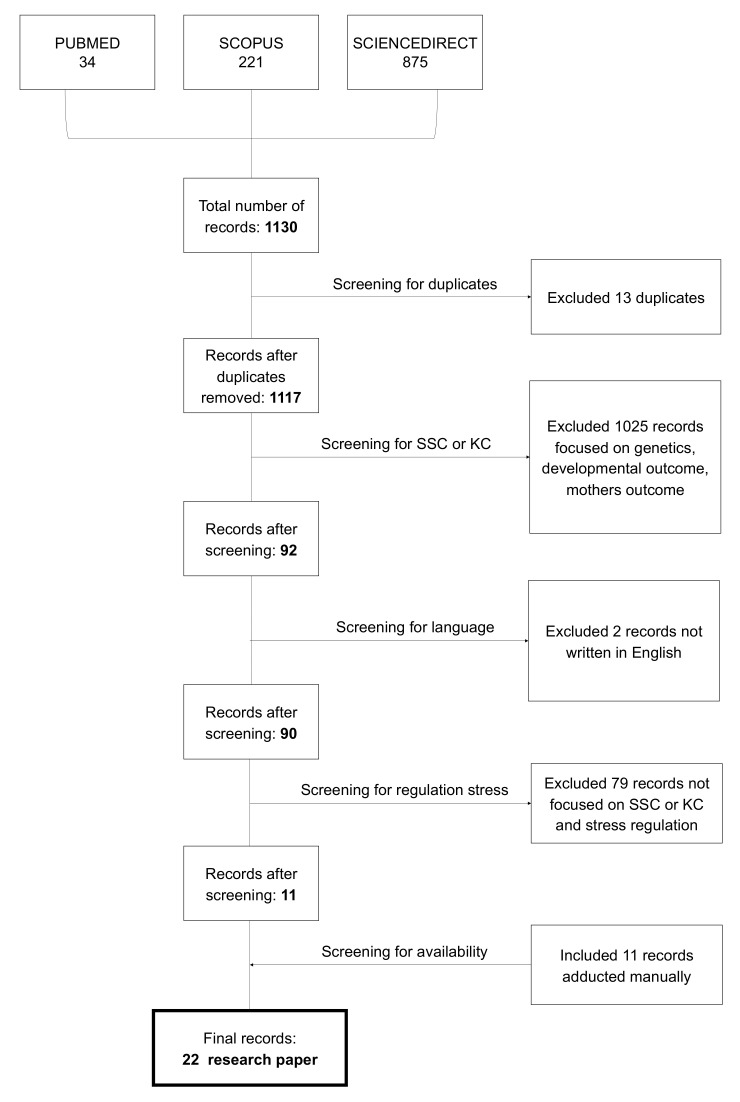
Flow diagram.

Articles that did not meet the criteria were removed from the review. The primary reason for elimination was the lack of focus on KC or SSC for stress regulation, as seen in Figure 1.

Our research question was answered by 22 articles (see Table 1).

According to the PRISMA guidelines, the assessment of eligibility for inclusion was carried out and subsequently discussed by the authors to finalize the agreement. 

## 3. Results

Our results demonstrate how stress regulation influences physiological parameters such as heart rate variability (HRV), cortisol, oxytocin, and stress levels.

Indeed, analysis of the 22 selected studies revealed that nine studies focused on HRV [36,38,44,45,46,47,48,50,51], six studies on stress [26,36,38,41,54,55], and nine on cortisol [40,42,43,51,52,54,55,56,57], while the remaining six focused on oxytocin [40,43,48,53,55,57]. The studies are summarized in Table 1.

### 3.1. Heart Rate Variability

HRV is a non-invasive measurement able to assess the ANS activity [58]. In the literature, studies have explained how high frequencies (over 0.15 Hz) could be related to parasympathetic activity [59], compared with low frequencies connected to both the parasympathetic and the sympathetic systems [60]. Various studies have investigated the effect of SSC on stress measured by HRV. Butruille et al. [36], in a study conducted in 2017, hypothesized that SSC could potentiate maternal and neonatal parasympathetic activities. They measured HRV by referring to two specific parameters: the Analgesia Nociception Index (ANI) for mothers and the Newborn Infant Parasympathetic Evaluation (NIPE) for infants. Results showed no difference in NIPE scores between SSC and incubator care. Indeed, infants with lower parasympathetic activity before SSC reacted with an increase in NIPE during SSC, whereas those with higher parasympathetic activity at baseline did not respond significantly [36]. Similar findings were presented by Harrison et al. [44,45] in two studies. In the first one [44], the researchers conducted a two-week feasibility trial of the effects of SSC on the ANS of infants with congenital heart disease (CCHD). HRV was used as a measure for the ANS functions. Furthermore, the researchers suggested improvements in ANS function with SSC and described nonlinear HRV measures connected with ANS for the first time. In the second study [45], they found that the parasympathetic activity in both the typically developing (TD) and cardiac SSC groups decreased.

In the research series published by Kommers et al. [47,48,49,50], different results were found. In one study, no statistically significant HRV was reported [47], and significantly lower HRV was reported in the other two studies [48,49]. In response to parental co-regulation, Kommers et al. [50] suggested that a decrease of the HRV parameters during SSC should be interpreted as a positive effect of SSC and an indication of better stability.

### 3.2. Maternal Stress

In a study conducted by Cho et al. [38], maternal stress and HRV were measured before and after KC practice. KC was performed three times per week, with women instructed to remove their shirts and sterilize their chest to welcome their babies. Results demonstrated a significant reduction in stress level after KC. This outcome was supported by the results of previous studies [61]. The authors explained this drop in stress level, more direct contact of mothers with their infants, adjustment to the NICU surroundings, better communication with medical teams during KC, and closer monitoring by babies’ mums throughout KC. Similar results were found in the study of Coskun et al. [41], where mothers who performed KC had lower levels on the parental stressor scale. 

### 3.3. Cortisol

SSC has been recognized to decrease stress reactivity in mothers and newborns. In particular, researchers have investigated salivary cortisol levels as a measure of cardiopulmonary stabilization and discovered a drop in post-birth cortisol levels of newborns treated with SSC [62]. An equal outcome was obtained in the study of El-Farrash et al. [42]. Day 7 salivary cortisol, measured as a stress response indicator for newborns, significantly decreased after KC compared with the conventional care group. This result demonstrated how early physical contact with the mother could impact the neuroendocrine pathways of infants. 

Hardin et al. [43] found a decrease in cortisol reactivity only in the recommended-use KC group, indicating that consistent early KC use may be associated with an increased regulation of stress. On the contrary, Mirnia and colleagues found no statistically significant differences in stress levels measured according to salivary cortisol between infants receiving 45 min of SSC with their fathers and infants receiving incubator care [52]. Morelius et al. [54] performed the first study reporting salivary cortisol outcomes of preterm infants and their mothers during SSC immediately after birth. Results showed that compared with the SC group, salivary cortisol reactivity was significantly lower in the SSC group. Similarly, Vitnner and colleagues [57] found, during a 60-min SSC intervention, a decrease of salivary cortisol compared with baseline parameters; this finding was consistent whether the mother or father provided SSC (*p* < 0.001). Additionally, in Varela et al.’s [56] study, fathers who held their baby in SSC for the first time showed a significant decline in physiological cortisol.

### 3.4. Oxytocin

Several studies focused on oxytocin as a neurobiological mechanism connected to stress regulation. Based on a previous study, Hardin and colleagues [43] hypothesized that KC would positively impact increasing oxytocin levels in both mothers and their full-term infants and decrease infant cortisol reactivity. The results demonstrated higher levels of mother–infant dyad oxytocin in the KC group compared with the control group. Previous studies have theorized a bio-mechanism as an explanation of oxytocin improvement after KC [63]. Cong et al. [40] conducted the first study reporting parental salivary oxytocin and cortisol levels during maternal and paternal SSC. The results showed a growth in maternal and paternal oxytocin levels during maternal/paternal SSC (M-SSC/P-SSC), but mothers and fathers expressed different oxytocin response patterns 30 min after SSC. Indeed, maternal oxytocin decreased after SSC, whereas paternal oxytocin continuously remained at a higher level [40]. Meanwhile, both maternal and paternal cortisol levels decreased significantly during SSC, and afterwards, maternal cortisol decreased continuously compared with paternal cortisol, which increased after P-SSC. Such results indicated that the oxytocinergic system can modulate parent–infant relationships through oxytocin release and that maternal behaviours can further strengthen the oxytocin system in both mother and infant [40].

## 4. Discussion

Tactile sensations are the most developed sensory pathways at birth [64]. In particular, mothers provide tactile stimulation through SSC and touch, including gentle massage. The literature demonstrated how SSC (or KC) could reduce parental stress and anxiety. However, the bio-behavioural mechanism involved in this process is still unknown [16].

This review aimed to explore and investigate how SSC regulates stress in an infant–parent relationship. Our results highlighted several biological mechanisms involved in parent–infant stress regulation, such as cortisol, HRV, and oxytocin.

Indeed, SSC between mothers and newborns is hypothesized to activate the infant and parent’s oxytocinergic system.

In the literature, the effects of M-SSC on stress reduction have been frequently measured in infants [65], but less research has studied the effects of P-SSC on fathers’ stress.

In Hardin et al.’s [43] study, for example, KC had moderate to significant effects on increasing oxytocin levels in mothers and infants at three months postpartum and decreasing cortisol reactivity in infants after acute stressor exposure. Identical results were obtained in the study of Cong et al. [40] and Vitnner et al. [57] for both maternal and paternal oxytocin levels. Indeed, Vitnner et al.’s study showed how parents’ lower cortisol and higher oxytocin levels activated during SSC sessions were significantly related to pre-discharge parental involvement [57]. Furthermore, rises in oxytocin levels and concurrent decreases in cortisol levels during SSC enable both infants and parents to develop a more synchronous relationship, improving bonding and attachment opportunities, especially in the challenging NICU environment [57]. In contrast, in Kommers et al.’s [49] study, a decline of oxytocin was found for preterm infants. This outcome could be explained by higher baseline oxytocin levels, which influenced a decrease in oxytocin during KC. Another explanation could be a switch in the regulation from the sympathetic nervous system’s dominance to that of the vagal systems [33]. Another crucial physiological parameter observed and connected with SSC and stress is the cortisol hormone. Indeed, it is well established that the HPA axis can control levels of cortisol, one of the most critical stress hormones. 

In the paraventricular nucleus (PVN) of the hypothalamus, corticotrophin-releasing hormone is produced and moved to the anterior pituitary, where it stimulates the release into the bloodstream of adrenocorticotrophic hormone (ACTH), which is responsible for cortisol’s secretion from the adrenal cortex via the activation of ACTH receptors [66]. The HPA axis and the ANS are the essential components of the stress system, specifically the SNS, also known as the fight or flight system [67]. The most commonly used and accepted indicator for evaluating the HPA axis functioning is cortisol hormone production.

For example, Varela et al. [56] discovered a reduction in paternal cortisol levels. The psychological effects of intimate contact between the father and baby when they were close together in SSC for the first time could explain this significant reduction in physiological stress. 

It is well known that premature babies’ fathers are usually exposed to a very high-stress level in the NICU [68,69]. In particular, previous studies found that holding their child in SSC leads to fathers feeling more in control of the situation and responding better to stressor events [70]. Another explanation could be that this first physical contact helps the fathers recover their role as parents and gives them a sense of control that could be very important for the future relationship [56].

The decrease in the secretion of cortisol by fathers in Varela’s study [56] was consistent with the previous research conducted by Cong et al. [40], which showed that 30 min of SSC with a very premature infant effectively reduced the levels of salivary cortisol in fathers.

Furthermore, SSC has also been shown to decrease salivary cortisol reactivity in babies and improve the concordance between mothers’ and infants’ salivary cortisol levels [54]. Indeed, Morelius et al. [54] found that mothers suffered a significant decrease in cortisol levels during their first SSC or after the SSC was over but not during the intervention. This outcome was confirmed by the study of Mirnia et al. [52], where in both skin-to-skin groups and standard ward treatment groups, cortisol in neonates decreased. In particular, compared with similar time intervals in the group with usual care, the cortisol level in neonates during SSC decreased more. Additionally, the infants experienced a comparable slope in the third time interval, in which the infants were separated from their fathers’ chest and received standard care, as did the control group [52].

Furthermore, in response to SSC, reduced sympathetic nervous activity is also involved in decreased heart rate and blood pressure induced by SSC.

Indeed, various studies have been conducted on SSC and heart rate variability. The dynamic, rapidly occurring changes in autonomic regulation caused by the primary HR control systems are reflected in the HRV. In addition to humoral factors, HR can be influenced immediately by the SNS and the PSNS [47]. Several characteristics to analyse these beat-to-beat changes were constructed and studied in adults, with at least some consensus on their interpretation. However, HRV in neonates has been less thoroughly researched. 

Additionally, the neonatal heart’s behaviour is significantly different from that of the adult heart, particularly in premature neonates, reflecting underlying autonomic regulation differences. 

This suggests that HRV-based characteristic interpretations in neonates may differ from those of adults [47]. In Kommers et al.’s [47] study, in contrast to what would have been expected in adults, low/high frequencies (LF/HF) increased during KC. The HF power decreased significantly, probably because the deceleration was less extreme, and fewer HF components were added to the total signal. These results and previous studies indicate that the LF/HF ratio cannot capture relaxation or sympathovagal balance in preterm infants [71]. Furthermore, Cho et al. [38] showed no significant differences in the physiological effects (weight, HR, body temperature, and oxygen saturation) between preterm infants who did and did not receive KC. 

These results are comparable to those previously obtained in the literature [72]. 

Other studies have shown different results [73]. After KC, the improved HR was due to the change in position and caused by the increased body temperature due to SSC of the infants with their mothers.

Researchers have not only found SSC to be safe in infants with CCHD but have reported improvements in physiological parameters in these particularly vulnerable infants [44].

Despite the advantages of the practice, SSC is not always prioritized by governments and clinicians.

In an observational study by Abdulghani et al. [74], a lack of adherence to SSC practice was presented together with SSC clinicians’ obstacles.

Additionally, due to concerns about the transmission of severe acute respiratory syndrome-coronavirus-2 (SARS-CoV-2), especially in the delivery room, the new coronavirus disease 2019 (COVID-19) epidemic represents the most recent and, at the same time, the most challenging obstacle for SSC. 

Indeed, the COVID-19 pandemic has caused unexpected abrupt changes in several neonatal care hospital practices, including delivery room management of mothers and infants. 

It is paradigmatic that during the COVID-19 emergency, SSC between the mother and her neonate is no longer permitted in many hospitals in Italy, the United States, and other countries worldwide.

However, in the literature, no significant concerns about SSC were raised during the 2009 H1N1 (swine flu) or the SARS and Middle East respiratory syndrome (MERS) epidemics, as no significant changes were advocated in infant feeding practices [75].

The worldwide recommendations on SSC were controversial at the outset of the current pandemic. 

Indeed, during the COVID-19 pandemic, SSC has sometimes been supported, but most of the time it is contraindicated or simply omitted [76,77].

The WHO has published detailed guidance recommending that infants and mothers with suspected or confirmed COVID-19 “should be enabled to remain together and practice skin-to-skin contact, kangaroo care and to remain together and to practice rooming-in throughout the day and night” [78].

In contrast, the Centers for Disease Control of the United States advised, “*consider separating the mother from her child temporarily*” until the mother is no longer considered contagious. Furthermore, it suggested that the healthcare team discuss “*the risks and benefits of temporary separation*” without more profound elaboration.

The advantage of separation is that during hospitalization, it minimizes the risk of COVID-19 transmission from mother to infant. However, if the goal is the health and well-being of both mother and child, additional factors must be considered.

First, the interruption of SSC disrupts newborns’ physiology. Children who are separated from their mothers have higher heartbeat rates and respiration and lower glucose levels than children who receive SSC [79]. As reported by the Royal College of Obstetricians and Gynecologists, “routine precautionary separation of a mother and a healthy baby should not be carried out lightly, given the possible harmful effects on feeding and bonding” [80].

Furthermore, isolation is a significant stressor for newborn infants and could worsen the condition’s progression for those infants previously infected with COVID-19. Additionally, for mothers, isolation could be perceived as a significant stressor. Indeed, in the NICU, during SSC, their HR, salivary cortisol levels, and stress scores decreased [81].

Separating mothers from their infants can cause significant suffering, particularly in the context of being diagnosed with a pandemic disease, and the associated physiological stress could worsen the mother’s condition. However, as reviewed here, the available evidence shows that maternal separation’s psychological outcomes are more significant than the potential harms of maternal SSC in terms of the proximity of SARS-CoV-2. Similar considerations have been undertaken by many governments, hospitals, and researchers that decided to keep mothers and infants together, supporting SSC.

Where policies or other circumstances prevent infants from being placed after birth in SSC, maintaining proximity to their mothers, or breastfeeding, health servers have an ethical duty to improve the advice [82].

## 5. Conclusions

The current evidence on the effects of SSC on stress regulation is summarized in this review. In this study, the results we reviewed reflect that SSC can regulate the stress, anxiety, and psychological distress of both the mother and the infant. 

During COVID-19, scientific societies are expected to legitimize and integrate SSC into their clinical practice indices based on new knowledge on the perinatal area and recently decided by the Italian Society of Neonatology [83]. 

Further steps towards its implementation will be needed after SSC is incorporated into clinical guidelines and protocols. In conclusion, considering that no data to date supports an increased risk of neonatal COVID-19 infection following SSC or delivery of a COVID-19-positive mother, it should be advised that SSC continue to be practiced as recommended by the WHO for all women [78].

## Figures and Tables

**Table 1:** Features of the studies included.

Study	Scope of the Study	Participants	Findings	Physiological Outcomes
Butruille, 2017 [36]	Influence of skin-to-skin contact (SSC) on the parasympathetic activity evaluated by heart rate variability (HRV)	Twenty-two infants and their mothers	SSC had a favourable impact on maternal and premature infant parasympathetic activities with a pronounced neonate response when baseline HRV values were lower	HRV
Carozza, 2020 [37]	Brief review of how contact influences the development of infant somatosensory, autonomic, and immune systems		Contact is an essential pathway for establishing and maintaining behavioural, physiological, and neural levels of parent–child interaction synchrony	Stress
Cho, 2016 [38]	Explore the effects of kangaroo care (KC) on physiological functions of preterm infants and maternal stress.	Twenty infants were assigned to the experimental group and 20 to the control group	KC had positive effects on stabilizing infant physiological functions such as respiration rate and reducing maternal stress.	HRV and maternal stress
Cleveland, 2017 [39]	Explain the effect of SSC on full-term newborns		Recommendations of SSC for healthy newborns	
Cong, 2015 [40]	Investigate how oxytocin modulates parental stress and anxiety during maternal and paternal SSC	Twenty-eight stable preterm infants and their parents	Both maternal and paternal oxytocin levels were significantly increased from baseline during the SSC. Both maternal and paternal cortisol levels decreased significantly from baseline during SSC.	Oxytocin and cortisol
Coskun, 2019 [41]	Investigate the effects of KC on the mother’s stress and amount of milk production	Eighty-four preterm newborns and their mothers	KC is effective in stimulating breast milk production and lowering maternal stress levels.	Maternal stress
El Farrash, 2019 [42]	Assess the effect of KC and its duration on the neurobehavioral system and salivary cortisol	One hundred and twenty stable preterm neonates	KC improved higher scores for regulation, non-optimal reflexes, and movement quality and lower scores for handling, excitement, and cortisol compared with the control group	Cortisol
Hardin, 2020 [43]	Examine EEG patterns along with basal oxytocin and cortisol reactivity in infants related to KC	Thirty-three mother–infant dyads at neonatal and three-month periods	KC increased oxytocin levels and decreased cortisol reactivity.	Oxytocin and cortisol
Harrison, 2017 [44]	Examine SSC’s effects on autonomic nervous system (ANS) functions	Eighteen infants and their mothers	HRV measures suggested improvements to the ANS functions after SSC	HRV
Harrison, 2019 [45]	Investigate the effects of SSC on learning and autonomic functions in three-month-old infants	Ten infants with congenital heart disease (CHD) who received neonatal SSC, 16 typically developing (TD) infants, and 10 infants with CCHD without SSC	The findings suggest improvements in cognitive and autonomic development in three-month-old CCHD infants who received neonatal SSC.	HRV
Jones et al. 2017 [46]	Explore the effect of SSC between parents and their neonates on parents’ heart rate (HR)	Twenty-six parents and their babies	A statistically significant difference between the parents’ initial HR and HR after SSC	HRV
Kommers, 2017 [47]	Explore whether HRV could be a measure to track regulatory changes during KC	Eleven preterm infants	A statistically significant difference in HRV between periods of KC and pre-KC	HRV
Kommers, 2018 [48]	Explore if a mattress that mimics breathing motion and heartbeat sounds can have the same effects as KC in preterm infants, as measured by HRV	Twenty preterm infants	HRV decreased during KC and after KC. No non-mattress effects were reported.	HRV
Kommers, 2018 [49]	Explore whether KC influences the salivary oxytocin concentration in preterm infants	Eleven twin pairs	Preterm infant twins’ oxytocin concentrations decreased during KC	Oxytocin
Kommers et al., 2019 [50]	Vital signs and HRV were analysed during KC with and without the use of a swaddling device to identify any potential changes	Twenty preterm infants	KC decreased heart rate, respiratory rate, and HRV. No changes were found regarding the swaddling device	HRV
Lisanti, 2020 [51]	Estimate SSC’s effect on mothers’ bio-behavioural stress measures (anxiety and salivary cortisol) before and after neonatal cardiac surgery	Thirty women and their infants	Significant reductions in self-reported anxiety and salivary cortisol scores were identified as a result of SSC.	Cortisol, HRV
Mirnia et al., 2017 [52]	Investigate the effect of SSC by fathers on salivary cortisol of infants	Forty-five premature infants and their fathers	SSC decreased levels of cortisol in babies.	Cortisol
Moberg, 2020 [53]	Describe the oxytocinergic system and the cutaneous sensory pathways activated by SSC		Decreased stress levels could be considered due to oxytocin’s ability to reduce the amygdala’s activity, the HPA-axis, and the sympathetic nervous system.	Oxytocin
Morelius, 2015 [54]	Investigate the effects of SSC on salivary cortisol, parental stress, depression, and breastfeeding	Thirty-seven families	SSC reduces infant cortisol reactivity in response to treatment and improves concordance between mother and infant salivary cortisol levels.	Cortisol and parental stress
Pados, 2019 [26]	Describe the physiological stress mechanisms that contribute to infant mortality and morbidity in the NICU and the physiological mechanisms by which SSC acts on the stress response system		Importance of SSC to mother–infant stress regulation	Stress
Pados et al., 2020 [55]	Investigate if SSC is an intervention used to reduce stress in the NICU		Research supports that SSC improves short-term cardiorespiratory stress outcomes compared with incubator care.	Stress, cortisol HRV, oxytocin
Varela, 2017 [56]	Explore paternal physiological stress during SSC with their babies	Forty-nine fathers	Fathers who practiced SSC showed a significant reduction in physiological stress outcomes.	Cortisol
Vitnner, 2018 [57]	Examine the relationship between parental involvement and salivary oxytocin and cortisol levels for parents participating in SSC intervention	Thirty-two stable preterm infants and their mothers and fathers	Significant relationships exist between parental engagement and salivary oxytocin and cortisol levels.	Oxytocin and cortisol

## Data Availability

Not applicable.
